# A Patient's Appreciation

**Published:** 1913-11-29

**Authors:** Edward A. Attwood

**Affiliations:** Secretary London Homœopathic Hospital


*To the Editor of* The Hospital.

### A Patient's Appreciation.

*To the Editor of* The Hospital.

Sir,—In these times of strenuous work to collect funds to maintain our voluntary hospitals it is refreshing to receive a letter of thanks like the following, the more especially as we shall be ending the year with a deficit on the income and expenditure account of the hospital of some £3,800 :—

To the Secretary, London Homœopathic Hospital.

November 18, 1913.

Sir,—I am sorry to say that it is quite beyond my power to endow either a cot at a cost of £750, or a bed at a cost of £1,000, in the hospital. When I wrote you I was under the impression that it cost about £200. I have lost my husband and have no child to carry on his name, and the idea occurred to me that I might endow a bed and so perpetuate his name, but this I regret is now impossible. Ten years ago I was a patient in the London Homœopathic Hospital, and received much kindness and benefit from my stay there. I have never forgotten the debt I owe the hospital, but it is only now that it has been in my power to do anything. Within a few months of my stay in the hospital I was widowed and had to return to school life. I enclose a cheque for two guineas as a subscription, and will send it each year until I am obliged to give up my position, and at my decease will bequeath £200 to the London Homœopathic Hospital. I am only sorry I cannot do more.—Yours faithfully,

A. M. H.

I am, yours faithfully,

Edward A. Attwood, Secretary.

London Homœopathic Hospital, November 25, 1913.

